# Noradrenergic Suppression of Persistent Firing in Hippocampal CA1 Pyramidal Cells through cAMP-PKA Pathway

**DOI:** 10.1523/ENEURO.0440-20.2020

**Published:** 2021-03-19

**Authors:** Maria Jesus Valero-Aracama, Antonio Reboreda, Alberto Arboit, Magdalena Sauvage, Motoharu Yoshida

**Affiliations:** 1 Faculty of Psychology, Mercator Research Group-Structure of Memory, Ruhr-University Bochum, Bochum 44780, Germany; 2 German Center for Neurodegenerative Diseases (DZNE), Magdeburg 39120, Germany; 3 Leibniz Institute for Neurobiology (LIN), Magdeburg 39118, Germany; 4 Otto von Guericke University, Functional Neuroplasticity, Medical Faculty, Magdeburg 39120, Germany; 5 Center for Behavioral Brain Sciences (CBBS), 39106, Magdeburg, Germany

**Keywords:** β1 adrenoceptors, cAMP, M1 receptor, M2/4 receptors, persistent firing, PKA

## Abstract

Persistent firing is believed to be a cellular correlate of working memory. While the effects of noradrenaline (NA) on working memory have widely been described, its effect on the cellular mechanisms of persistent firing remains largely unknown. Using *in vitro* intracellular recordings, we demonstrate that persistent firing is supported by individual neurons in hippocampal CA1 pyramidal cells through cholinergic receptor activation, but is dramatically attenuated by NA. In contrast to the classical theory that recurrent synaptic excitation supports persistent firing, suppression of persistent firing by NA was independent of synaptic transmission, indicating that the mechanism is intrinsic to individual cells. In agreement with detrimental effects of cAMP on working memory, we demonstrate that the suppressive effect of NA was through cAMP-PKA pathway. In addition, activation of β1 and/or β3 adrenergic receptors, which increases cAMP levels, suppressed persistent firing. These results are in line with working memory decline observed during high levels of NA and cAMP, which are implicated in high stress, aging, and schizophrenia.

## Significance Statement

While cholinergic modulation supports working memory, high concentrations of noradrenaline (NA), which occurs under high stress for example, are detrimental for working memory. However, cellular and molecular mechanisms underlying such working memory deficit remain largely unclear. In this paper, we studied the effect of these two neuromodulators on persistent firing, the cellular correlate of working memory. We demonstrate that a cholinergic receptor activation supports, while a noradrenergic activation strongly inhibits persistent firing because of the PKA activation through specific NA receptor types which upregulate cAMP. These data are in line with working memory deficits in aging and schizophrenia in which cAMP levels are altered, and indicate potential intrinsic cellular mechanism of working memory impairment.

## Introduction

Persistent firing is a cellular response characterized by repetitive spiking that outlasts triggering stimulus, and is believed to be a neural base of working memory (Goldman-Rakic, 1995; Major and Tank, 2004). Persistent firing observed in humans and animals *in vivo* during working memory tasks correlate with the task performance (Funahashi et al., 1989; Colombo and Gross, 1994; Hampson and Deadwyler, 2003; Bukalo et al., 2004; Kamiński et al., 2017). In contrast to the classical hypothesis that persistent firing is supported by recurrent excitatory synaptic connections, recent studies have shown that persistent firing can also be supported in individual cells (Egorov et al., 2002; Jochems and Yoshida, 2013; Knauer et al., 2013). These studies in general used cholinergic receptor activations to induce persistent firing, which is in agreement with the supportive role of acetylcholine on working memory (Reboreda et al., 2018). However, roles of other neuromodulators such as the noradrenaline (NA) on this type of persistent firing largely remain to be studied.

While cholinergic modulation generally supports working memory (Kaneko and Thompson, 1997; Weiss et al., 2000), NA could be detrimental for working memory (Arnsten, 2009; Roozendaal and McGaugh, 2011). High levels of NA are usually present during states of stress (Bremner et al., 1996), which are known to impair working memory (Diamond et al., 1996; Schoofs et al., 2008; Qin et al., 2009; Arnsten et al., 2012). At the receptor subtype levels, β1 and α2 adrenoreceptors, which are positively and negatively coupled to cAMP signaling, impair and facilitate working memory, respectively (Jäkälä et al., 1999; Li et al., 1999; Ma et al., 2003; Arnsten and Li, 2005; Ramos et al., 2005). In addition, elevated and decreased cAMP levels impair and enhance, respectively, working memory performance (Arnsten et al., 1999; Taylor et al., 1999; Runyan et al., 2005; Wang et al., 2007). Moreover, upregulated cAMP levels are associated with working memory impairment in aging and schizophrenia (Millar, 2005; Wang et al., 2011; Barch and Ceaser, 2012). Raising cAMP levels would increase PKA activation that also is negatively correlated to working memory performance (Kobori et al., 2015), together supporting the importance of NA and cAMP-PKA in working memory.

At the cellular level, a supportive role of NA on persistent firing through a reduction of cAMP levels has been observed in the prefrontal cortex (PFC; Zhang et al., 2013). In addition, elevated cAMP levels caused a suppression of the calcium-activated nonspecific cation (CAN) current and persistent firing in the PFC and hippocampus (Sidiropoulou et al., 2009; El-Hassar et al., 2011; Zhang et al., 2013). However, it remains unclear whether NA has a supportive or detrimental effect on persistent firing in the hippocampus.

We therefore tested the effects of cholinergic and noradrenergic receptor activation in both induction and modulation of persistent firing in individual hippocampal CA1 pyramidal cells in mice. We find that a noradrenergic stimulation strongly inhibits persistent firing while a cholinergic activation supports it. In addition, the noradrenergic suppression of persistent firing is independent of ionotropic synaptic transmission, but is through the cAMP-PKA pathway. We further demonstrate specific NA receptor subtypes involved in this suppression. These observations indicate that high NA and cAMP conditions, which often suppress working memory through PKA activation, also suppress cellular mechanisms for persistent firing in the hippocampus.

## Materials and Methods

All the experimental designs were approved by the local ethic committee (Der Tierschutzbeauftragte, Ruhr-Universität Bochum, and Deutsches Zentrum für Neurodegenerative Erkrankungen) and conducted in accordance with the European Communities Council Directive of September 22, 2010 (2010/63/EU).

### Slice preparation

Acute hippocampal slices were obtained from adult (two to four months) C57BL6 female mice (Charles River). Animals were deeply anesthetized (intraperitoneal injection of 120 mg/kg of ketamine and 16 mg/kg of xylazin) and intracardiac perfusion with ice-cold modified artificial CSF (mACSF) was conducted directly without or after cervical dislocation. The mACSF contained 87 mm NaCl, 25 mm NaHCO_3_, 10 mm glucose, 75 mm sucrose, 2.5 mm KCl, 1.25 mm NaH_2_PO_4_, 0.375 mm CaCl_2_, 3.28 mm MgCl_2_, 3 mm pyruvix acid, and 1 mm ascorbic acid or 87 mm NaCl, 25 mm NaHCO_3_, 10 mm glucose, 75 mm sucrose, 1.25 mm KCl, 1.25 mm NaH_2_PO_4_, 0.188 mm CaCl_2_, 1.64 mm MgCl_2_, 3 mm pyruvix acid, and 1 mm ascorbic acid (pH was adjusted to 7.4 by saturation with 95% O_2_-5% CO_2_). Following the decapitation, horizontal hippocampal slices (350 μm) were obtained with a vibrating-blade microtome (Leica VT1000S) in ice-cold mACSF. Brain slices were then incubated for 30 min at 37°C in normal ACSF (nACSF) containing 124 mm NaCl, 1.25 mm NaH_2_PO_4_, 1.8 mm MgO_4_S, 3 mm KCL, 10 mm glucose, 26 mm NaHCO_3_, and 1.2 mm CaCl_2_ or 124 mm NaCl, 1.25 mm NaH_2_PO_4_, 1.8 mm MgO_4_S, 1.5 mm KCL, 10 mm glucose, 26 mm NaHCO_3_, and 0.6 mm CaCl_2_ (pH was adjusted to 7.4 by saturation with 95% O_2_-5% CO_2_). The second nACSF with 1.5 mm KCL was used in an attempt to reduce synaptic effect but was used only in seven cells in which ICI-118,551 was applied. In all other 105 cells, the first nACSF with 3 mm KCL was used. Brain slices were maintained at room temperature for at least 30 min before recording.

**Figure 5.**
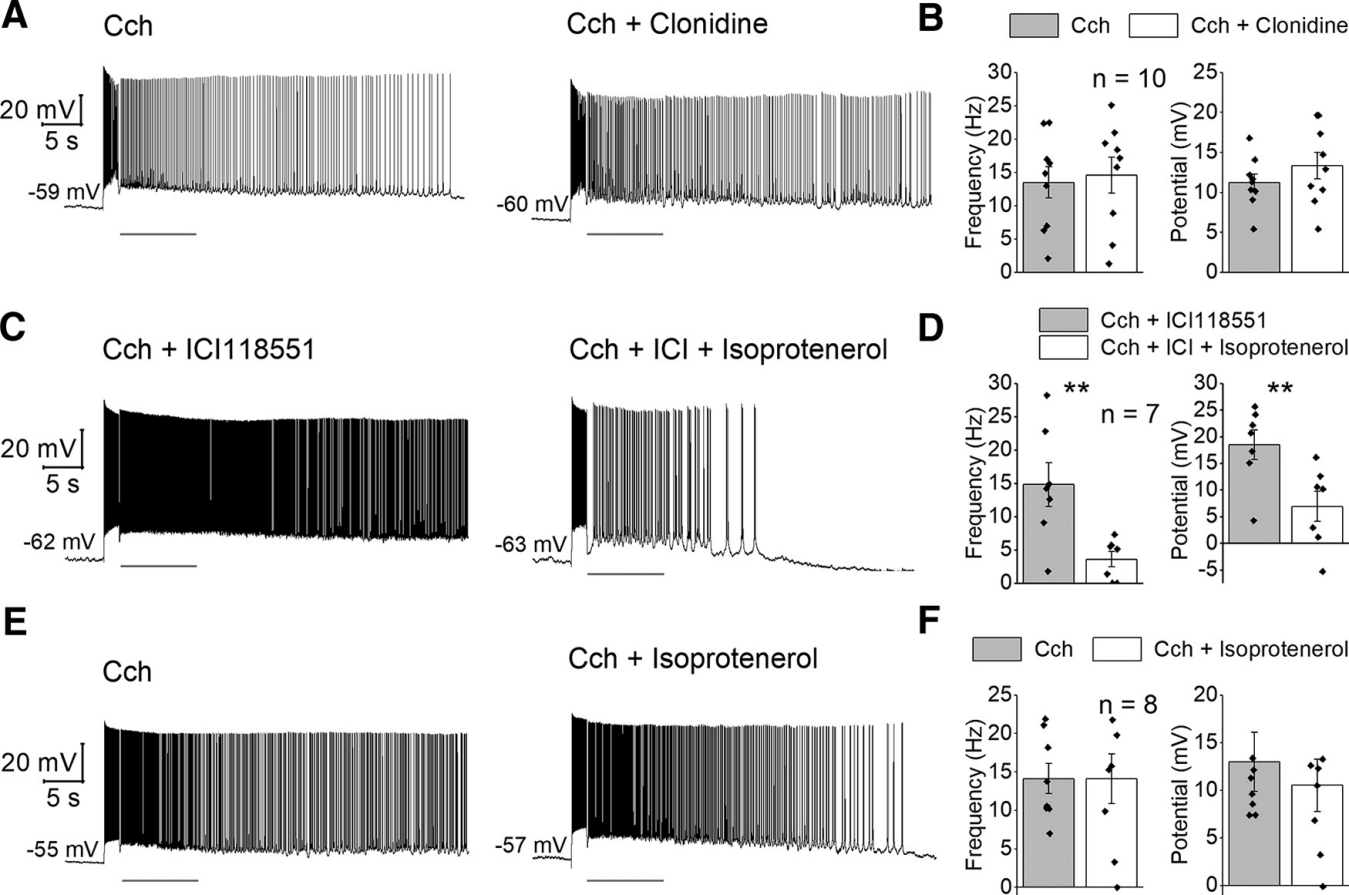
Roles of NA receptor subtypes in suppression of persistent firing. ***A***, Representative traces of the effect of clonidine on the cholinergic persistent firing. ***B***, Poststimulus frequency (left) and poststimulus potential (right) in Cch (gray) and Cch and clonidine (white). ***C***, Representative traces of the effect of β2 (inhibited by ICI) versus β1 and β3 adrenergic receptors (activated by isoprotenerol in the presence of ICI) on the cholinergic persistent firing. ***D***, Poststimulus frequency (left) and poststimulus potential (right) in a cocktail of Cch and ICI (gray) and in a cocktail of Cch, ICI and ISO (white). ***E***, Representative traces of the effect of β-receptor stimulation by isoprotenerol. ***F***, Poststimulus frequency (left) and poststimulus potential (right) in (gray) and Cch and ISO (white).

### Whole-cell recordings

Whole-cell procedures were performed as described previously (Valero-Aracama et al., 2015). Briefly, CA1 cells were visually identified using an upright microscope (Zeiss Axioskop 2FS), and whole-cell patch was obtained using glass pipettes filled with an intracellular solution containing 120 mm Kgluc, 10 mm HEPES, 0.2 mm EGTA, 20 mm KCl, 2 mm MgCl_2_, 7 mm PhCreat di(tris), 4 mm Na_2_ATP, and 0.3 mm Tris GTP (pH adjusted to 7.3 with KOH). Electrical signals were amplified with an Axoclamp 2A amplifier (Molecular Devices), low-pass filtered at 10 kHz and sampled at 20 kHz, using WinWCP (John Dempster, University of Strathclyde) software. Liquid junction potential (∼10 mV) was not corrected. To prevent pharmachological contamination, only one cell per slice was recorded. Recordings were performed at 34 ± 1°C.

### Drugs

All drugs except for carbachol (Cch; Alfa Aesar) were purchased from Sigma-Aldrich. Water-based stock solutions were made for all drugs except picrotoxin and kynurenic acid (freshly dissolved in nACSF). Except for the Cch stock solution, which was stored at 4°C, all other solutions were aliquoted and stored at −20°C. Stock solutions were diluted at least 1000 times in the final solution. Recording solutions with Cch were freshly made each day of experiment and solutions containing other drugs were freshly made from stock solutions minutes before being used. Recordings started at least 5 min after the application of the experimental drug.

### Data analysis

Analysis was conducted with MATLAB R2009b (MathWorks) and Anaconda (Python 3.6). Only pyramidal cells with a silent resting membrane potential lower than −55 mV, and spike amplitudes that overshoot 0 mV were included into the analysis. Persistent firing was induced by a current injection (2 s, 100 pA) from a membrane potential below spiking threshold. The membrane potential and frequency of persistent firing were measured during the first 10s after the termination of the stimulation. General excitability was measured from the current-frequency curve (50–350 pA, 50-pA increment, 1 s) starting from the fix potential of −65 mV. Input resistance (IR) was computed from the voltage drop peak in response to a 50-pA negative current injection from −65 mV. Sag ratio was computed from voltage response to a negative current step (1 s, −300 pA). Medium and slow after-hyperpolarizing potential (AHP) were measured after the induction of six spikes (0.1–1 nA, 3 ms, 50 Hz) from a membrane potential of −60 mV. In cases the AHP was positive compared with the baseline (as in conditions with Cch) this property is referred to as ADP. Comparisons were made using paired *t* tests. Significance level α < 0.05 (*p < 0.05, **p < 0.01, ***p < 0.001) was used. Data are expressed as mean ± SEM.

## Results

### Cch supports persistent firing through G_q/11_ and G_i/o_ G-protein-coupled receptors (GPCRs)

**Figure 1.**
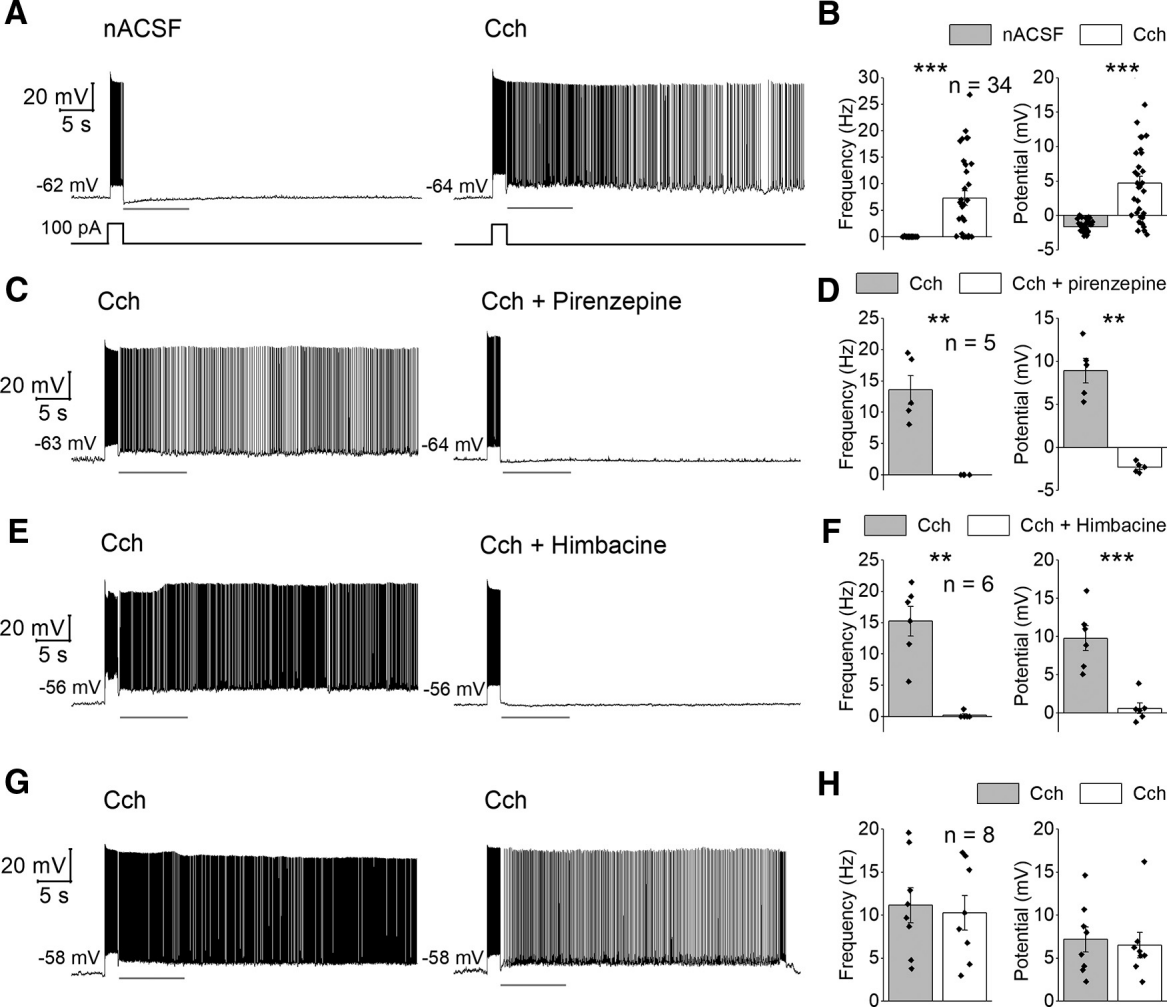
Cholinergic induction of persistent firing in CA1 hippocampal neurons. ***A***, Representative cell’s responses in nACSF (left) and in Cch (right). The gray line indicates the 10-s period in which characteristics of persistent firing were analyzed. Trace at the bottom shows the current step applied to test persistent firing. ***B***, Poststimulus frequency (left) and poststimulus potential (right) in nACSF (gray) and Cch (white). ***C***, Representative trace of the effect of muscarinic M1 antagonist pirenzepine on the cholinergic persistent firing. ***D***, Poststimulus frequency (left) and poststimulus potential (right) in Cch (gray) and Cch and pirenzepine (white). ***E***, Representative trace of the effect of muscarinic M2/M4 antagonist himbacine on the cholinergic persistent firing. ***F***, Poststimulus frequency (left) and poststimulus potential (right) in Cch (gray) and Cch and himbacine (white). ***G***, Representative responses from timed control experiment. ***H***, Poststimulus frequency (left) and poststimulus potential (right) in the first test in Cch (gray) and the second test in Cch (white).

First, we tested the effects of cholinergic stimulation alone on the generation of persistent firing in CA1 pyramidal cells. Once a whole-cell recording was obtained, induction of persistent firing was tested by using a brief (2 s, 100 pA) current injection at a membrane potential right below the spike threshold. In the nACSF, none of the cells tested continued spiking after the termination of the stimulation (*n* = 34; Fig. 1*A*, left). However, in the presence of Cch (5 μm), persistent firing was induced in 62% (21 out of 34) of the same set of cells (Fig. 1*A*, right). This is in agreement with previous reports of similar persistent firing in rat and mouse CA1 pyramidal cells in Cch (Knauer et al., 2013; Arboit et al., 2020). For the quantification of persistent firing, spike frequency and membrane potential after the stimulation were measured: poststimulus frequency and poststimulus potential. The gray line below each voltage trace indicates the period during which these measurements were taken. Cch significantly increased the poststimulus frequency (*n* = 34; *T*_(33)_ = −5.65, *p* < 0.001; Fig. 1*B*, left) and the poststimulus membrane potential (*n* = 34; *T*_(33)_ = −6.67, *p* < 0.001; Fig. 1*B*, right).

Cch activates both G_q/11_ and G_i/o_ GPCRs through M1 and M2/4 receptor subtypes, respectively. To study the role of each receptor subtype on persistent firing, we selectively blocked M1 or M2/4 receptors. Bath application of the M1 antagonist pirenzepine (10 μm), in addition to Cch, resulted in a complete blockade of persistent firing (Fig. 1*C*,*D*). The poststimulus frequency (*n* = 5; *T*_(4)_ = 5.94, *p* < 0.01; Fig. 1*D*, left) and depolarization (*n* = 5; *T*_(4)_ = 7.10, *p* < 0.01; Fig. 1*D*, right) were significantly reduced. Similarly to this, M2/4 antagonist himbacine (1 μm) suppressed persistent firing significantly (Fig. 1*E*) reducing both the frequency (*n* = 6; *T*_(5)_ = 6.59, *p* < 0.01; Fig. 1*F*, left) and the depolarization (*n* = 6; *T*_(5)_ = 7.83, *p* < 0.001; Fig. 1*F*, right). These results point out that both G_q/11_-coupled and G_i/o_-coupled receptors are necessary to trigger persistent firing. Furthermore, a control experiment was conducted to exclude the possibility that the suppressive effect of pirenzepine and himbacine was because of a run-down of cellular mechanisms supporting persistent firing during the whole-cell recording. In this experiment, persistent firing recorded at two different time points after the break-in was compared in the presence of Cch. The first and second time points were comparable to the times at which persistent firing was tested in Cch alone and Cch + pirenzepine (or himbacine), respectively, in the above experiment. Persistent firing was not reduced, as shown in Figure 1*G*,*H* (frequency: *n* = 8, *T*_(7)_ = 0.388, *p* = 0.709; potential: *n* = 8, *T*_(7)_ = 0.730, *p* = 0.489), indicating that the suppression of persistent firing was not an artifact of our experimental procedure.

### NA does not support persistent firing

**Figure 2.**
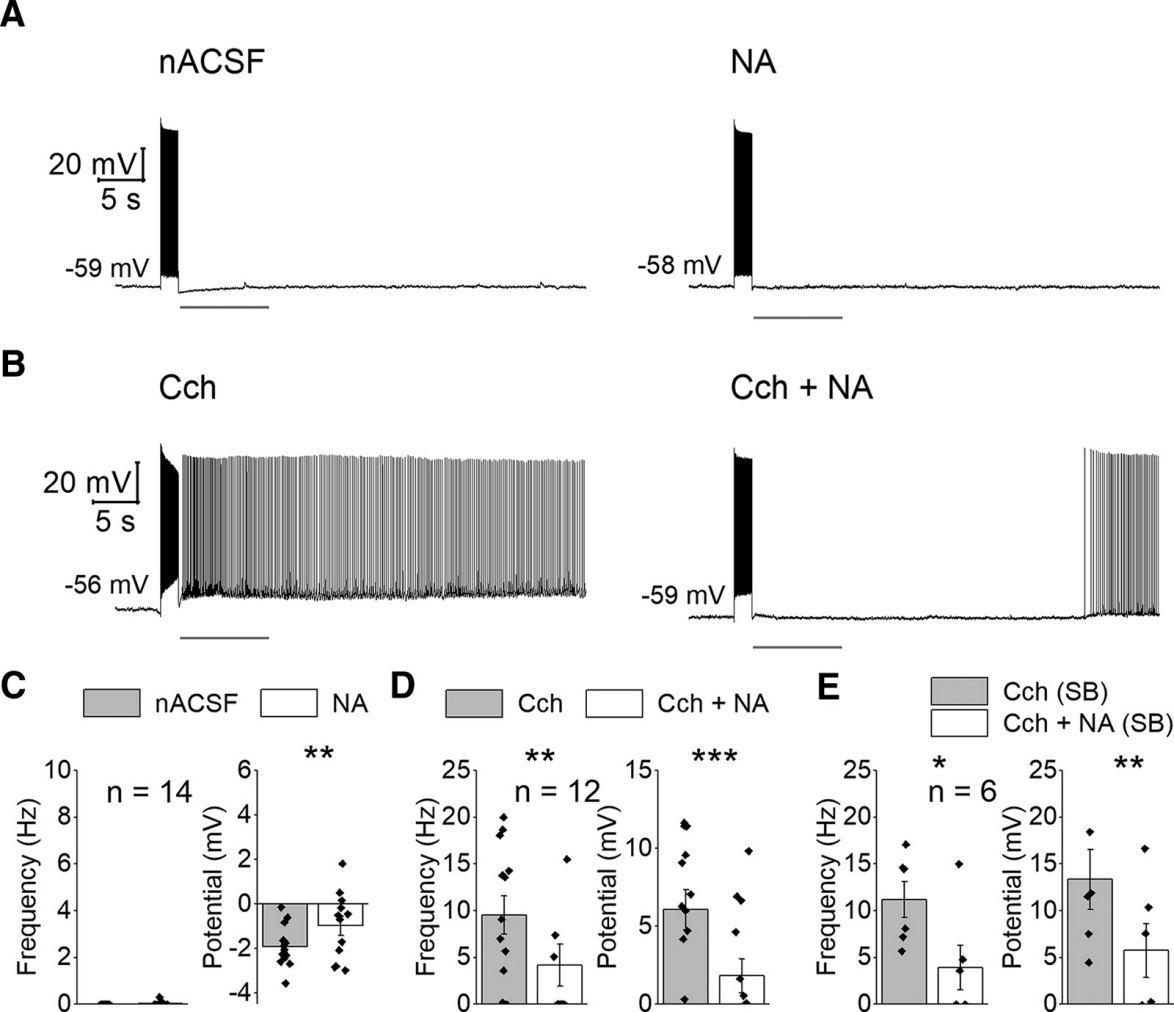
NA suppresses persistent firing. ***A***, Representative traces of the effect of NA in nACSF. ***B***, Representative traces of the effect of NA on the cholinergic persistent firing. ***C***, Effect of NA on poststimulus frequency (left) and poststimulus potential (right) without Cch. ***D***, Same as in ***C*** but in Cch. ***E***, Same as in ***D*** but in the presence of the SBs.

We next tested the effects of NA (5–10 μm; *n* = 14) on the induction of persistent firing. Persistent firing was not observed in any of the cells recorded and the poststimulus firing frequency was unchanged (Fig. 2*A*). While the poststimulus membrane hyperpolarization (AHP) was reduced by NA (*n* = 14; *T*_(13)_ = −3.068, *p* = 0.009; Fig. 2*C*), the membrane potential did not reach levels above the baseline (averaged voltage before the stimulation) unlike Cch.

It has been reported that intrinsic excitability of CA1 pyramidal cells is increased by both cholinergic and noradrenergic modulation (Benardo and Prince, 1982; Madison and Nicoll, 1982). In agreement with these, the numbers of spikes elicited during the stimulation (2 s, 100 pA) were significantly larger in both Cch and NA compared with those in the nACSF (Cch: nACSF 23 ± 1, Cch 31 ± 2, *n* = 34, *T*_(33)_ = −4.69, *p* < 0.001; NA: nACSF 21 ± 1, NA 28 ± 2, *n* = 14, *T*_(13)_ = −4.77, *p* < 0.001). A reduced AHP mentioned above is also in agreement with the previous report (Madison and Nicoll, 1982), while the mAHP measured after five spikes (Table 1) did not show significant decrease probably because of a bad signal-to-noise ratio.

**Table 1:** Effect of Cch and NA on general cellular properties

Part I								
		50 pA	100 pA	150 pA	200 pA	250 pA	300 pA	350 pA
nACSF	*n *=* *47	1 ± 0	11 ± 2	21 ± 2	31 ± 2	37 ± 2	42 ± 2	46 ± 2
Cch		6 ± 2**	23 ± 2***	35 ± 2***	41 ± 2***	46 ± 2***	49 ± 2***	53 ± 3**
nACSF	*n *=* *13	1 ± 1	7 ± 3	15 ± 3	22 ± 4	27 ± 5		
NA		2 ± 1	12 ± 3*	23 ± 4**	31 ± 4**	36 ± 4**		

Part II										
	IR (MΩ)		TH (mV)		SAG		mAHP (mV)		ADP (mV)	
	*n*		*n*		*n*		*n*		*n*	
nACSF	48	105 ± 4	51	−47.9 ± 0.6	48	0.166 ± 0.01	42	−1.16 ± 0.11	42	−0.27 ± 0.07
Cch		139 ± 6***		−48.9 ± 0.7**		0.151 ± 0.01		−0.26 ± 0.14***		1.24 ± 0.15***
nACSF	15	90 ± 6	9	−46.4 ± 2.0	15	0.173 ± 0.01	9	−1.47 ± 0.34	9	−0.40 ± 0.25
NA		115 ± 8***		−47.3 ± 1.8		0.115 ± 0.01**		−1.33 ± 0.27		0.23 ± 0.13*

Part I, Number of spikes elicited by 1-s current steps. Part II, Input resistance (IR), spike threshold (TH), sag ratio (SAG), medium AHP (mAHP), after depolarizing potential (ADP). Values correspond to mean ± SEM. Significance level: *p < 0.05, **p < 0.01, ***p < 0.001.

**Table 2:** Effect of NA on top of Cch on general cellular properties

Part I								
		50 pA	100 pA	150 pA	200 pA	250 pA	300 pA	350 pA
Cch	*n *=* *13	6 ± 3	26 ± 4	37 ± 3	41 ± 3	47 ± 3	50 ± 3	53 ± 3
Cch + NA	19 ± 5	29 ± 5	42 ± 4	48 ± 4	54 ± 5	57 ± 6	60 ± 5	

Part II						
		IR (MΩ)	TH (mV)	SAG	mAHP (mV)	ADP (mV)
Cch	*n *=* *13	142 ± 11	−51.0 ± 1.6	0.147 ± 0.02	−0.14 ± 0.29	1.28 ± 0.26
Cch + NA	129 ± 10*	−53.7 ± 1.6	0.145 ± 0.01	−0.27 ± 0.22	0.84 ± 0.12	

Part I, Number of spikes elicited by 1-s current steps. Part II, Input resistance (IR), spike threshold (TH), sag ratio (SAG), medium AHP (mAHP), after depolarizing potential (ADP). Values correspond to mean ± SEM.

These indicate that persistent firing was not simply the result of increased excitability because persistent firing was observed only in Cch. This is in line with the specific role of the CAN current in support of persistent firing, which is activated downstream of the cholinergic receptor (Yan et al., 2009; Zhang et al., 2011; Knauer et al., 2013). Changes of other basic properties by Cch and NA are listed in the Table 1.

### Effect of NA on cholinergically-induced persistent firing

Based on the observation that NA alone did not support the induction of persistent firing, we next tested whether NA has modulatory effects on persistent firing under cholinergic activation. In this experiment, persistent firing was tested first in nACSF, then in Cch (5 μm), and finally in a solution containing Cch and NA (5–10 μm). To ensure a full effect of NA, persistent firing was tested ∼15 min after the initiation of the NA application. In ten neurons that showed persistent firing in Cch condition, persistent firing was completely blocked in six, suppressed in three, and slightly increased in one cell by NA (Fig. 2*B*). Poststimulus firing frequency and membrane potential were both significantly decreased by NA (frequency: *n* = 12, *T*_(11)_ = 4.118, *p* = 0.002; potential: *n* = 12, *T*_(11)_ = 5.647, *p* < 0.001; Fig. 2*D*,*E*). Cells that did not exhibit persistent firing in Cch did not show it in NA either (*n* = 3; data not shown).

Next, to assess whether the reduction of persistent firing was synaptic or intrinsic, the effect of NA on cholinergically induced persistent firing was examined in the presence of synaptic blockers (SBs) that blocked the ionotropic excitatory and inhibitory transmissions (0.1 mm picrotoxin and 2 mm kynurenic acid). In line with our previous study in rat CA1 pyramidal cells (Knauer et al., 2013), induction of persistent firing was not affected by the presence of the SBs, indicating that persistent firing is supported by intrinsic cellular mechanisms (data not shown). As for the modulation of persistent firing, an application of NA suppressed persistent firing significantly similarly to the case without SBs (frequency: *n* = 6, *T*_(5)_ = 3.748, *p* = 0.013; potential: *n* = 6; *T*_(5)_ = −6.016, *p* = 0.002; Fig. 2*E*). Together, these data indicate that suppression of persistent firing was probably caused by other mechanisms than a potential change in synaptic transmission because of NA. Changes of other basic properties by NA in the presence of Cch are listed in the Table 2.

### Intracellular mechanisms underlying the suppression of persistent firing

Cholinergic and noradrenergic systems act through different receptors coupled to mainly three types of GPCRs. While Ach activates only G_q/11_ (M1, M3, M5) and G_i/o_ (M2, M4) pathways, NA can activate G_s_ (β1, β2, β3) in addition to G_q/11_ (α1) and G_i/o_ (α2, partially β2) pathways. The G_s_ pathway, unlike other two pathways, increases the cAMP level, which is detrimental to working memory (Dash et al., 2007). In addition, an elevated cAMP level has been shown to suppress persistent firing *in vivo* in the PFC, and suppresses the CAN current in the hippocampus as mentioned above (El-Hassar et al., 2011). Therefore, we tested whether NA suppressed persistent firing by a cAMP activation through the G_s_ pathway.

**Figure 3.**
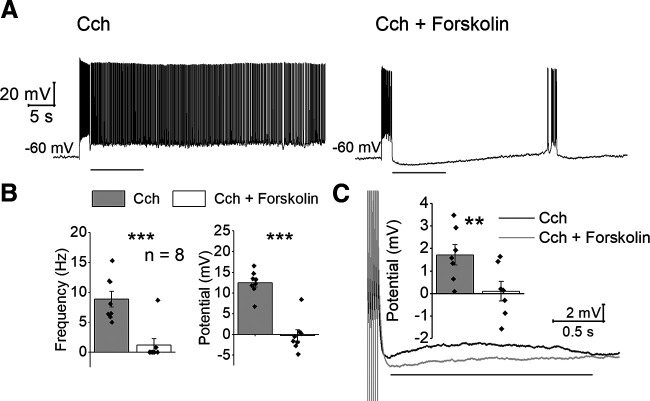
Forskolin suppresses persistent firing. ***A***, Representative traces of the inhibition by forskolin on the cholinergic persistent firing. ***B***, Poststimulus frequency (left) and poststimulus potential (right) in Cch (gray) and Cch+forskolin (white). ***C***, Representative after depolarization following six spikes in Cch (gray) and in a cocktail containing Cch and forskolin (gray). Gray straight line indicates the section analyzed. Vertical lines under truncated spikes indicate the timing of current injection. The bar graph shows the membrane potential in Cch (gray) and Cch+forskolin (white).

**Figure 4.**
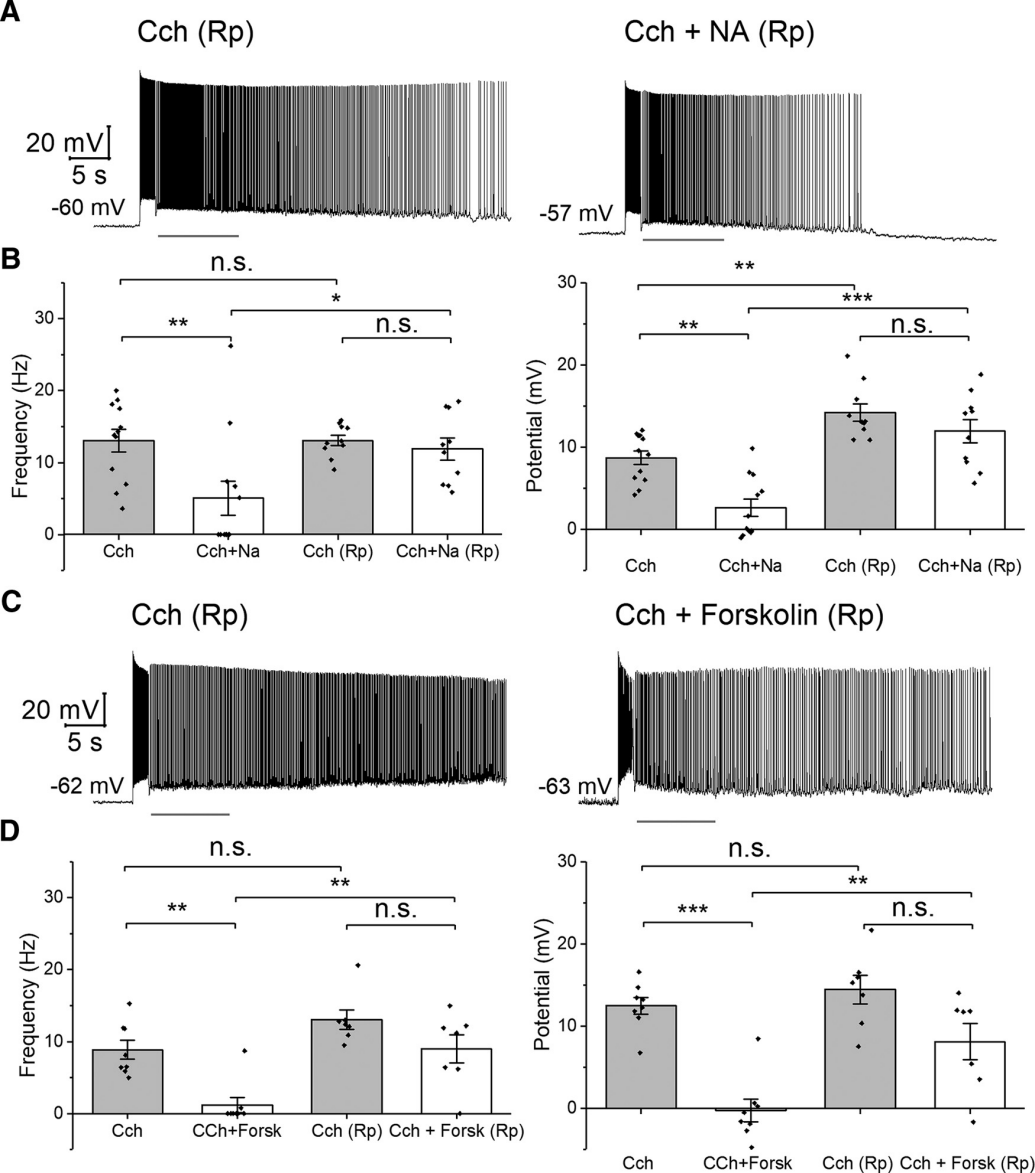
Inhibition of PKA by Rp-cAMPS blocks the NA and forskolin mediated reduction of persistent firing. ***A***, Representative traces of the effect of Rp-cAMPS on the NA-mediated inhibition of the cholinergic persistent firing. ***B***, Poststimulus frequency (left) and poststimulus potential (right) comparison with two-way repeated-measures ANOVA followed by Tukey’s *post hoc* test. The first two columns show measurements without Rp-cAMPS from previous experiments. ***C***, Representative traces of the effect of Rp-cAMPS on the forskolin-mediated inhibition of the cholinergic persistent firing. ***D***, Poststimulus frequency (left) and poststimulus potential (right) comparison with two-way repeated-measures ANOVA followed by Tukey’s *post hoc* test.

First, we evaluated the effect of an elevated cAMP concentration on persistent firing using forskolin, a compound that activates the adenylate cyclase and therefore increases intracellular levels of cAMP. Persistent firing was first tested in Cch (5 μm) and then forskolin (10 μm) was bath applied in addition to Cch. This and other experiments in this section were conducted in the presence of SBs. Forskolin clearly suppressed persistent firing (frequency: *n* = 8, *T*_(7)_ = 8.165, *p* < 0.001; potential: *n* = 8, *T*_(7)_ = 8.165, *p* < 0.001; Fig. 3*A*,*B*). We found that forskolin also decreased the number of spikes during the stimulation (Cch: 23.56 ± 1.5; Cch+forskolin: 19 ± 1.8; *n* = 8; *T*_(7)_ = 3.789, *p* = 0.007). To study whether the suppression of persistent firing was simply because of the smaller number of spikes elicited during the stimulation, response of the cells to a fixed number (six) of induced spikes were additionally tested (Fig. 3*C*). Spikes were induced by using six brief current injections (0.1–1 nA, 3 ms, 50 Hz) at −60 mV, and the poststimulus potential was measured as an average membrane depolarization during the 2-s period after the simulation offset (gray line). The poststimulus potential was significantly smaller in the Cch+forskolin condition compared with that in Cch alone (*n* = 7; *T*_(6)_ = 4.803, *p* = 0.003; Fig. 4*C*), suggesting that the suppression of persistent firing by forskolin was not simply because of the reduced number of spikes triggered during the stimulation.

The fact that forskolin is able to suppress persistent firing might be indicating that NA suppression could be generated by an increase of cAMP that leads to PKA activation. To test this hypothesis, we included the competitive PKA antagonist Rp-cAMPS (100 μm) in the recording pipette. In this condition, NA mediated inhibition of persistent firing was strongly reduced (*n* = 10; Fig. 4*A*). Figure 4*B* shows the effect of NA on the frequency and depolarization of persistent firing with and without Rp-cAMPS. Rp-cAMPS prevented the suppressive effect of NA on persistent firing, maintaining persistent firing similar to that in the control condition without NA (Fig. 4*B*). A multiple comparison with a two-way repeated-measures ANOVA followed by pairwise comparisons with the Tukey’s *post hoc* test indicated that neither the frequency or depolarization changed significantly when Rp-cAMPS was present (the third and fourth columns), while both were reduced significantly by NA when Rp-cAMPS was not present (the first and second columns). In contrast, when persistent firing in NA was compared with or without Rp-cAMPS (the second and fourth columns), both frequency and depolarization were significantly larger when Rp-cAMPS was present (frequency: *t*_(20)_ = 4.104, *p* = 0.040; potential: *t*_(20)_ = 8.932, *p* < 0.001; Fig. 4*B*). These indicate that suppression of persistent firing by NA is mainly by G_s_ activation of PKA.

As a control experiment, we repeated the forskolin application in the presence of Rp-cAMPS (Fig. 4*C*). Repeated-measures two-way ANOVA coupled with the Tukey’s *post hoc* test indicated that forskolin inhibition of persistent firing was strongly reduced in the presence of Rp-cAMPS (frequency: *t*_(13)_ = 3.164, *p* = 0.164; potential: *t*_(13)_ = 1.321, *p* = 0.787; Fig. 4*D*, third and fourth columns). In contrast, Rp-cAMPS had a significant effect on the frequency and depolarization in the presence of forskolin (frequency: *t*_(13)_ = 5.903, *p* = 0.005; potential: *t*_(13)_ = 5.660, *p* = 0.007; Fig. 4*D*, second and fourth column). These indicate that the suppressive effect of forskolin was through PKA activation.

Experiments with forskolin and Rp-cAMPS supported the idea that NA interfered with persistent firing through a cAMP mediated activation of PKA. To further demonstrate the involvement of receptor subtypes coupled to the G_s_ pathway, effects of individual receptor agonists were tested. First, the effect of the α2 receptor agonist clonidine (10 μm), which decreases cAMP levels through G_i/o_ activation, was examined in the presence of Cch. As expected, clonidine did not inhibit persistent firing (frequency: *n* = 10, *T*_(9)_ = −0.646, *p* = 0.534; potential: *n* = 10; *T*_(9)_ = 1.234, *p* = 0.252; Fig. 5*A*,*B*). As for the adrenergic β receptors, although all β receptors (β1, β2, β3) are coupled to G_s_, recent studies pointed out that β2 activates the G_i_ in addition to the G_s_ cascade (Hall, 2004; Schutsky et al., 2011a,b). Therefore, we aimed to activate β1 and β3 receptors without activating β2 by using the general β receptor agonist isoproterenol (ISO; 1 μm) along with the β2 blocker ICI-118,551 (ICI; 1 μm) based on a previous report (Schutsky et al., 2011a). Combination of ISO and ICI significantly inhibited persistent firing (frequency: *n* = 7, *T*_(6)_ = 4.634, *p* = 0.004; potential: *n* = 7, *T*_(6)_ = 4.488, *p* = 0.004; Fig. 5*C*,*D*). In contrast, a sole application of the general β agonist ISO did not affect persistent firing either at 1 μm (frequency: *n* = 7, *T*_(6)_ = 1.788, *p* = 0.124; potential: *n* = 7, *T*_(6)_ = 0.777, *p* = 0.467) or at 10 μm (frequency: *n* = 8, *T*_(7)_ = 0.008, *p* = 0.994; potential: *n* = 8, *T*_(7)_ = 0.729, *p* = 0.128; Fig. 5*E*,*F*), indicating that, in line with previous literature, β2 might induce actions that counteract the increase of cAMP. In summary, these results suggest that NA suppresses cholinergically-induced persistent firing via PKA activation downstream of a β1 and/or β3 receptors mediated increase of cAMP.

## Discussion

Based on the differential modulation of working memory by acetylcholine and NA, we investigated effects of these neuromodulators on the induction and modulation of persistent firing in hippocampal CA1 pyramidal cells in brain slices taken from adult female mice. Results from this study indicate that only the cholinergic agonist Cch, but not NA, is able to support induction of persistent firing. However, when NA was applied in addition to Cch, NA strongly suppressed cholinergically triggered persistent firing. We have further shown that the suppression of persistent firing by NA was not a result of decreased intrinsic excitability or ionotropic synaptic transmissions. In contrast, forskolin, the specific activation of β1 and PKA blockade with Rp-cAMPS indicated that cAMP elevations through the G_s_-protein cascade activating PKA caused the suppression of the cholinergic persistent firing.

### Molecular mechanisms underlying suppression of persistent firing

Persistent firing similar to our study is shown to be supported by intrinsic cellular mechanisms in multiple brain areas involved in working memory (Fraser and MacVicar, 1996; Haj-Dahmane and Andrade, 1998; Egorov et al., 2002; Tahvildari et al., 2008; Navaroli et al., 2012; Jochems and Yoshida, 2013; Knauer et al., 2013). These studies have pointed out that this type of persistent firing is supported by the CAN current, which is believed to be supported by TRPC channels (Reboreda et al., 2011; Zhang et al., 2011; Arboit et al., 2020). In line with our data, the CAN current and TRPC channels are inhibited by the cAMP (Partridge et al., 1990; El-Hassar et al., 2011; Sung et al., 2011). In particular, El-Hassar et al. (2011) used forskolin in the hippocampal CA1 pyramidal cells as in our study to indicate that TRPC mediated CAN current is suppressed. Suppression of similar persistent firing by cAMP was also reported in the entorhinal cortex and PFC (Sidiropoulou et al., 2009; Zhang et al., 2013). Therefore, CAN current suppression is a feasible mechanism for the suppression of persistent firing in our study.

However, it is possible that other mechanisms were additionally involved. One possibility could be that an increase in GABAergic input mediated by NA (Kawaguchi and Shindou, 1998; Sessler et al.,, 1995) suppressed persistent firing. Inhibitory synaptic input is known to be higher in the presence of NA (Kawaguchi and Shindou, 1998; Sessler et al., 1995). Inhibitory synaptic input is effective in terminating persistent firing in the dentate gyrus (Anderson and Strowbridge, 2014). This interpretation was in line with the decreased IR we observed in NA, which could be caused by the opening of GABAergic receptors. To gain insight into this, we used the same SBs as in Zhang et al. (2013), which demonstrated facilitated persistent firing by NA in PFC neurons. However, in the presence of the SBs, IR was still reduced and persistent firing was suppressed by NA. Based on these, we suggest that changes in synaptic transmission were not the main cause of persistent firing suppression in our study.

Another possibility is that the elevation of cAMP has modulated the hyperpolarization-activated cyclic nucleotide-gated (HCN) channels. In the PFC, adrenergic stimulation of α2 receptors improved working memory performance by suppressing HCN channels through cAMP downregulation (Wang et al., 2007). This is in line with facilitated persistent firing in the PFC neurons under the blockade of HCN channels (Zhang et al., 2013). This mechanism might, however, not be involved in the suppression of persistent firing in our study because the sag potential, which indicates the amount of HCN current, was rather reduced by NA, suggesting a reduction, if any, of HCN current. In addition, α2 receptor agonist clonidine did not reduce the sag potential in our study. This could be because (1) the cAMP levels were already low because of Cch, and α2 activation could not further reduce the cAMP level; (2) modulation of HCN channels by cAMP is pronounced in young but is weak in adult animals in the hippocampus (Surges et al., 2006); and (3) the α2 receptor does not modulate the HCN channels because co-localization of the α2 receptor with the HCN channels, which is reported in the PFC (Wang et al., 2007), does not occur in the hippocampal CA1 pyramidal cells.

In addition, we cannot completely exclude the possibility that mGluR is involved in the suppression of persistent firing by NA. Similarly to Cch, mGluRs activate the G_q_ (group 1) and G_i_ (groups 2 and 3) pathways. When Cch is not present, NA-induced mGluR activation could play a role in modulating persistent firing as was the case in Zhang et al. (2013). However, in the presence of Cch as in our study, G_i_ and G_q_ are already highly activated, and an additional activation of the same G_i_ and G_q_ through mGluRs, if exists, could be marginal. This point has been tested by Zhang et al. (2013) by applying the mGluR blocker MPEP on persistent firing induced by Cch, and they showed no change of persistent firing, indicating that the role of mGluR in persistent firing is limited when Cch is present. In contrast, when mGluRs are the main activator of the G_q_ and G_i_ pathways, NA may have a similar suppressive effect on persistent firing in the hippocampus. This view is supported by the suppression of mGluR-induced “plateau potential” and persistent firing by cAMP and PKA in the hippocampus and PFC (Sidiropoulou et al., 2009; El-Hassar et al., 2011).

### Suppressed persistent firing as a possible cellular mechanism of impaired working memory in high NA levels

Roles of cholinergic receptor activation on persistent firing supported by intrinsic cellular mechanisms have been studied intensively *in vitro* (for review, see Major and Tank, 2004; Yoshida et al., 2012). Similarly to these prior studies, persistent firing was observed under cholinergic receptor activation in our experiment in hippocampal pyramidal CA1 cells in mice. This is in line with the supportive role of cholinergic activation on working memory (Kaneko and Thompson, 1997; Weiss et al., 2000). In addition, we have shown that cholinergically-induced persistent firing is suppressed by NA. In line with these results are the detrimental effects of high concentrations of NA on working memory (Arnsten, 2009; Roozendaal and McGaugh, 2011). Together, these observations indicate that both supportive (cholinergic) and detrimental (NA) modulations on working memory are in agreement with the cellular level modulation of PF, which is a possible cellular correlate of working memory.

Interestingly, persistent firing in our study was suppressed by β1/3 receptors activation (Fig. 5*D*), while the use of general β receptor agonist did not alter the response (Fig. 5*E*), suggesting that receptor subtypes have different effects on persistent firing. Traditionally, it has been believed that the actions of different β receptors were identical, all activating the G_s_ cascade. However, recent studies have suggested that β2 receptors might act through different mechanisms than β1, possibly by activating the G_i_ cascade in addition to the G_s_ cascade (Hall, 2004; Schutsky et al., 2011a,b). Since G_i_ cascade counteract the action of G_s_ cascade by decreasing cAMP levels, unclear effect of the general β receptor agonist (Fig. 5*E*) could have been resulted from this action of β2 receptors. Interestingly, the role of β receptors in working memory was tested using generic agonists or antagonists, resulting in no apparent effect on this cognitive function (Arnsten et al., 1999). On the other hand, the specific activation of β1 receptors has been shown to impair working memory, while the activation of β2 has improved it (Ramos et al., 2005, 2008). Therefore, modulations of persistent firing and working memory agree with each other at the level of receptor subtypes in this case.

Our data using Rp-cAMPS and forskolin further suggested that the suppression of persistent firing was through cAMP upregulation, which can be induced by the activation of the G_s_ cascade. Interestingly, multiple studies have indicated that increased cAMP levels impair working memory, while a reduction of cAMP levels improves it (Taylor et al., 1999; Ramos et al., 2003; Runyan et al., 2005). Moreover, increased cAMP reduced persistent firing *in vivo* during working memory tasks (Vijayraghavan et al., 2007; Wang et al., 2007). It is relevant to note that Rp-cAMPS has been tested *in vivo* in monkeys during an oculomotor delayed response working memory task (Vijayraghavan et al., 2007). In this study, Rp-cAMPS rescued D1 receptor mediated reduction of neural firing while an application of Rp-cAMPS alone increased the neuronal firing (Vijayraghavan et al., 2007). The rescue effect of Rp-cAMPS is in line with the suppressive roles of cAMP on persistent firing observed in our study. In addition, increased neural activity by the application of Rp-cAMPS *in vivo* is in line with the increased depolarization during persistent firing we observed in our study (Fig. 4*B*). In summary, G_s_-mediated cAMP upregulation seems to be detrimental for both working memory and persistent firing, while G_i_-mediated cAMP downregulation seems to support both working memory and persistent firing.

### Supportive role of moderate levels of NA on working memory

In contrast to the detrimental effect of high levels of NA, it has been proposed that moderate concentrations of NA aid working memory through the α2 receptors because of relatively high affinity of NA to the α2 receptors (Arnsten and Li, 2005). In fact, working memory is facilitated by the α2 receptor activation in several studies (Jäkälä et al., 1999; Li et al., 1999; Birnbaum et al., 2000; Ma et al., 2003; Arnsten and Li, 2005). In the present study, we tested two doses of NA (5 and 10 μm); however, we did not observe facilitative effect of NA on persistent firing either with or without cholinergic agonist Cch. The lack of facilitative effect of NA on persistent firing could be because the two concentrations tested were both high doses equivalent to situations of stress, where β1/3 receptors were active in addition to the α2 receptors, masking the positive effect of the α2 receptors. However, our experiment with the α2 receptor agonist indicates that there is no room for facilitation of persistent firing through α2 receptor activation at least in the presence of cholinergic agonist, meaning that in this condition, even a lower concentration of NA would not have facilitated persistent firing. The α2 receptor activates the G_i_ cascade, which decreases cAMP levels. One possibility is that Cch at the concentration we used already decreased cAMP levels to a minimum through G_i_ activation and therefore additional activation of the α2 receptors could not lower the cAMP levels further. In contrast to our study, in the rat PFC, application of NA (10 μm) enhanced persistent firing with or without the cholinergic agonist Cch (Zhang et al., 2013). In their study, the persistent firing facilitation was mediated by a combination of α1 and α2 receptors, and no regulation was observed by the β receptor activation. While the direction of modulation of persistent firing by NA is opposite from our study, the effect of cAMP level is consistent in both studies. While increased cAMP (through β receptors) suppressed persistent firing in our study, a decreased cAMP (through α2 receptors) facilitated persistent firing in Zhang et al. (2013). Therefore, the opposite effect of NA could be caused by activation of different receptor subtypes in these studies, possibly because of different affinities or expression density between the hippocampus and PFC. Although data available on these issues is limited, it has been reported that the β3 receptors are expressed more in the hippocampus than in the cortex, while α2 and β1 receptors are similarly expressed in these areas in rats (McCune et al., 1993; Nicholas et al., 1993a,b; Summers et al., 1995). Inhibitory effect of NA in our study might be linked to a stronger activation of G_s_ and cAMP upregulation through β3 receptors in the hippocampal neurons.

In conclusion, working memory impairment and compromised persistent firing have often been attributed to a reduced recurrent synaptic excitation (Wang, 2001; Arnsten, 2011). For example, it is proposed that activation of HCN channels and SK channels at dendritic terminals attenuates synaptic currents and reduce persistent spiking under cAMP elevation (Wang et al., 2007, 2011). On the other hand, our study together with recent studies (Sidiropoulou et al., 2009; El-Hassar et al., 2011) support the idea that cAMP may modulate working memory through direct inhibition of the neuronal ability to support persistent firing, possibly through the modulation of CAN/TRPC current.

Impaired working memory because of high levels of cAMP may also be involved in normal aging, schizophrenia and traumatic brain injury in addition to high stress conditions (Arnsten, 2011; Wang et al., 2011; Kobori et al., 2015). In support of this view, knocking down the disrupted in schizophrenia 1 (DISC1) protein (a protein that participates in cAMP catabolism) results in a decrease of the TRPC current in rat PFC neurons (El-Hassar et al., 2014). In addition, an infusion of Rp-cAMPS in traumatic brain injury or aged rats with working memory deficits, improves the performance on those tasks (Ramos et al., 2003; Kobori et al., 2015). Therefore, the mechanism of suppression of intrinsic persistent firing we present here might be relevant to working memory impairment in different conditions such as aging, traumatic brain injury and schizophrenia as well.

## Synthesis

Reviewing Editor: Liset Menendez de la Prida, Instituto Cajal CSIC Decisions are customarily a result of the Reviewing Editor and the peer reviewers coming together and discussing their recommendations until a consensus is reached. When revisions are invited, a fact-based synthesis statement explaining their decision and outlining what is needed to prepare a revision will be listed below. The following reviewer(s) agreed to reveal their identity: David Fernandez de Sevilla.

You manuscript was reviewed by two referees and myself and we all agree it has merits for publication. We found the modulation effect by noradrenaline novel and potentially interesting. The fact that it is opposite to that described in prefrontal cortex, suggests very interesting regional differences. However, we have raised a number of concerns and suggestions you may need to address in extent. Here is a synthesis of the comments:

Some major commments are:

1) It is unclear why two different solutions were used for cutting and incubating/recording. Specifically, authors use 3 mM KCL / 1.2 mM CaCl2 or 1.5 mM KCl / 0.6 mM CaCl2, but it is unclear in which conditions each solution was used. That could impact on the persistent firing as intrinsic excitability is dependent on the extracellular concentration of potassium and divalent ions. I would recommend authors testing this effect.

2) In the same vein, why the extracellular concentration of calcium was low (especially 0.6 mM Ca2+)? It would make sense to avoid synaptic transmission (balancing the concentration of divalent ions by increasing Mg2+), but since authors also used synaptic blockers it is unclear the impact of this manipulation. This should be addressed or clarified.

3) The suppression of the persistent firing by noradrenaline or increasing cAMP with forskolin was quite clear. However, the application of beta-adrenergic agonists (ICI + isoproterenol) did not have a strong effect. Could it be associated to some actions dependent on the beta-2 receptor pathway? Could authors address this point more specifically?

4) All intrinsic properties were measured at -65 mV except for the slow and medium AHP which were measured at -60 mV. I wonder if that holding membrane potential could be quite depolarized and some of the currents involved (such as M-current) could be partially opened. That would explain the small magnitude reported for the medium AHP and poor statistical effect of noradrenaline. I would consider running the analysis again for a more hyperpolarized holding potential resulting in larger AHP. Perhaps that could help to improve the SN ratio for more appropriate interpretation of pharmacological effects.

5) CA1 pyramidal neurons receive synaptic inputs via activation of metabotropic glutamate receptors (mGluRs) that could play an important role when neurons are firing due to the action of CCh. Although authors use blockers of the ionotropic receptors, the manuscript will have stronger impact by showing that the described NA modulation is not altered when blocking the mGluRs.

In addition the following comments should be considered:

6) Please, clarify at which temperature were recordings obtained.

7) The authors state that suppression of persistent firing was probably caused by intrinsic factors as the blockage of synaptic transmission with picrotoxin and kynurenic did not alter that suppression (and indeed they pharmacologically demonstrate this point in last figures). Could it be possible that part of the suppression was due to a synaptic modulation of the cholinergic transmission by noradrenaline? Please, clarify.

8) Since the authors show that that NA suppresses the cholinergically induced persistent firing via PKA downstream of the activation of β1 and/or β3 receptors this should appear in the abstract. In the present abstract, the authors only mention de activation of β1 adrenergic receptors.

9) The authors should define the meaning of PFC when it appears the first time in the introduction
